# The Myelin Content of the Human Precentral Hand Knob Reflects Interindividual Differences in Manual Motor Control at the Physiological and Behavioral Level

**DOI:** 10.1523/JNEUROSCI.0390-20.2021

**Published:** 2021-04-07

**Authors:** Raffaele Dubbioso, Kristoffer Hougaard Madsen, Axel Thielscher, Hartwig Roman Siebner

**Affiliations:** ^1^Danish Research Centre for Magnetic Resonance, Centre for Functional and Diagnostic Imaging and Research, Copenhagen University Hospital Hvidovre, 2650 Hvidovre, Denmark; ^2^Department of Neurosciences, Reproductive Sciences and Odontostomatology, University Federico II of Naples, 80131 Naples, Italy; ^3^Department of Applied Mathematics and Computer Science, Technical University of Denmark, 2800 Kgs. Lyngby, Denmark; ^4^Department of Health Technology, Technical University of Denmark, 2800 Kgs. Lyngby, Denmark; ^5^Department of Neurology, Copenhagen University Hospital Bispebjerg, 2400 Copenhagen, Denmark; ^6^Institute for Clinical Medicine, Faculty of Health and Medical Sciences, University of Copenhagen, 2200 Copenhagen, Denmark

**Keywords:** functional cortical mapping, magnetic resonance imaging, motor skill, premotor cortex, primary motor cortex

## Abstract

The primary motor cortex hand area (M1_HAND_) and adjacent dorsal premotor cortex (PMd) form the so-called motor hand knob in the precentral gyrus. M1_HAND_ and PMd are critical for dexterous hand use and are densely interconnected via corticocortical axons, lacking a sharp demarcating border. In 24 young right-handed volunteers, we performed multimodal mapping to delineate the relationship between structure and function in the right motor hand knob. Quantitative structural magnetic resonance imaging (MRI) at 3 tesla yielded regional R1 maps as a proxy of cortical myelin content. Participants also underwent functional MRI (fMRI). We mapped task-related activation and temporal precision, while they performed a visuomotor synchronization task requiring visually cued abduction movements with the left index or little finger. We also performed sulcus-aligned transcranial magnetic stimulation of the motor hand knob to localize the optimal site (hotspot) for evoking a motor evoked potential (MEP) in two intrinsic hand muscles. Individual motor hotspot locations varied along the rostrocaudal axis. The more rostral the motor hotspot location in the precentral crown, the longer were corticomotor MEP latencies. “Hotspot rostrality” was associated with the regional myelin content in the precentral hand knob. Cortical myelin content also correlated positively with task-related activation of the precentral crown and temporal precision during the visuomotor synchronization task. Together, our results suggest a link among cortical myelination, the spatial cortical representation, and temporal precision of finger movements. We hypothesize that the myelination of cortical axons facilitates neuronal integration in PMd and M1_HAND_ and, hereby, promotes the precise timing of movements.

**SIGNIFICANCE STATEMENT** Here we used magnetic resonance imaging and transcranial magnetic stimulation of the precentral motor hand knob to test for a link among cortical myelin content, functional corticomotor representations, and manual motor control. A higher myelin content of the precentral motor hand knob was associated with more rostral corticomotor presentations, with stronger task-related activation and a higher precision of movement timing during a visuomotor synchronization task. We propose that a high precentral myelin content enables fast and precise neuronal integration in M1 (primary motor cortex) and dorsal premotor cortex, resulting in higher temporal precision during dexterous hand use. Our results identify the degree of myelination as an important structural feature of the neocortex that is tightly linked to the function and behavior supported by the cortical area.

## Introduction

The primary motor cortex hand area (M1_HAND_) and adjacent dorsal premotor cortex (PMd) are critical for dexterous hand use in human and nonhuman primates. The M1_HAND_ enables the independent use of single fingers through direct cortico-motoneuronal control of hand and finger muscles (Lemon, 2008, 2019). The rostral (“old”) and caudal (“new”) parts of M1_HAND_ differ with respect to their cortico-motoneuronal connectivity (Rathelot and Strick, 2006, 2009; Witham et al., 2016). Only the caudal M1_HAND_, which is located deep in the anterior wall of the precentral sulcus contains large, fast-conducting pyramidal cells that produce short-latency monosynaptic responses in spinal motoneurons. The rostral part of M1_HAND_ (and the somatosensory area 3a) exert descending motor control over cervical spinal motoneurons via more slowly conducting monosynaptic as well as disynaptic projections (Witham et al., 2016). The PMd also plays an important role in manual motor control, contributing to the selection and execution of hand and finger movements (Picard and Strick, 2001; Ward et al., 2010). Its prominent role is reflected by dense reciprocal connections with M1_HAND_ (Dum and Strick, 2005) and by the fact that the most caudal part of PMd contains scattered large pyramidal cells that send axonal projections to the cervical cord via the pyramidal tract (Geyer et al., 2000).

The M1_HAND_ and PMd form a characteristic knob-like structure in the human precentral gyrus (Yousry et al., 1997). The precentral motor hand knob can be easily identified by structural magnetic resonance imaging (MRI) because of its visible Ω or ε shape (Yousry et al., 1997). The M1_HAND_ and PMd lack a clear anatomic demarcation line. Cytoarchitectonic mapping studies showed that the rostral border of the primary motor cortex extends to the surface of the precentral crown close to the midline, but retracts to the rostral bank of the central sulcus in more lateral parts of the hemisphere (Geyer et al., 1996, 2000). The caudal PMd occupies most of the crown and lip of the precentral hand region and belongs cytoarchitectonically to Brodmann area 6 (BA6). However, the transition between rostral M1_HAND_ and caudal PMd is smooth, and there is subject interindividual variability regarding the rostral extension of the M1_HAND_ (Geyer et al., 1996, 2000). In agreement with a smooth cytoarchitectonic transition, the dendritic tree of supragranular (layer III) pyramidal cells in the precentral cortex of macaques becomes gradually more complex with rostral progression from the central sulcus (M1) to adjacent premotor cortex (Elston and Rockland, 2002).

The physiology of the precentral motor hand knob and its corticomotor output to the contralateral hand can be studied in humans with transcranial magnetic stimulation (TMS; Barker et al., 1985; Palmer and Ashby, 1992; Maertens De Noordhout et al., 1999; Groppa et al., 2012). The site to target M1_HAND_ is functionally defined as the site where TMS elicits the largest motor evoked potential (MEP) in contralateral hand muscles, commonly referred to as “motor hotspot” (Groppa et al., 2012; Rossini et al., 2015). But TMS can also be used to map the functional topography of corticomotor representations by applying TMS at different scalp positions using a two-dimensional grid (Wassermann et al., 1993; Pascual-Leone et al., 1994; Veldema et al., 2017). TMS-based corticomotor mapping has consistently shown substantial interindividual variations in precentral motor hotspot location along the anterior–posterior (A–P) grid axis (Teitti et al., 2008; Diekhoff et al., 2011; Vaalto et al., 2011; Sarfeld et al., 2012; Ahdab et al., 2014, 2016).

Postmortem myeloarchitectonic analyses have shown that the precentral gyrus is one of the cortical areas that contains the highest density of myelinated axons (Nieuwenhuys, 2013). Using myelin-sensitive readouts (Glasser and van Essen, 2011; Lutti et al., 2014), *in vivo* MRI confirmed that the myelin content in the frontal cortex peaks in the pericentral gyrus and then gradually decreases along a posterior–anterior (P–A) gradient (Glasser and van Essen, 2011). MRI-based cortical myelin mapping also revealed considerable interindividual variability (Shams et al., 2019) with respect to the regional myelin content. Since myelination enables fast signal propagation and synchronizes neural activity (Seidl et al., 2010; Pajevic et al., 2014; Ford et al., 2015; Timmler and Simons, 2019), the degree of precentral myelination may account for functional phenotypic variation in precentral cortical function and dexterous hand use. To test this hypothesis, we combined structural myelin-sensitive MRI, task-related functional MRI, with a novel sulcus-aligned TMS mapping approach. We reasoned that our multimodal mapping approach can reveal structural and functional features in the precentral gyrus that may account for interindividual variability regarding the evoked corticomotor responses and contribute to interindividual differences in the plasticity-inducing effects of repetitive TMS on corticomotor excitability.

## Materials and Methods

### 

#### Participants and power analysis

Our main goal was to detect a link between regional cortical myelination and a read-out of corticomotor representation as revealed by our novel TMS mapping procedure (see below for details). Since this novel TMS-based measure (i.e., rostrality index) had not been used in previous work, we could not perform a proper power analysis. We therefore based our sample size estimation on previous neurophysiological TMS mapping studies. Here, the number of participants included in a single study ranged from 11 to 17 healthy volunteers (Teitti et al., 2008; Diekhoff et al., 2011; Vaalto et al., 2011, 2016; Sarfeld et al., 2012; Ahdab et al., 2014, 2016). We decided to recruit 24 participants to secure a sample size that exceeded previous TMS mapping studies, factoring in an estimated drop-out rate of 20%.

Twenty-four healthy young adults (12 women, 12 men) with a mean age of 24 years (age range, 19–34 years) and a mean height of 173 cm (height range, 163–187 cm) participated in this study. Participants were consistently right handed, as assessed by the Edinburgh handedness inventory (Oldfield, 1971). Only individuals with little (<2 years) or no formal music training were included. They had no history of neurologic or psychiatric disorders and were screened for contraindications to TMS (Rossi et al., 2009). They all gave written informed consent to the experimental procedures. The study complied with the Helsinki Declaration on human experimentation and was approved by the Ethical Committee of the Capital Region of Denmark (H-15000551).

#### Experimental procedures and data acquisition

**Figure 1.**
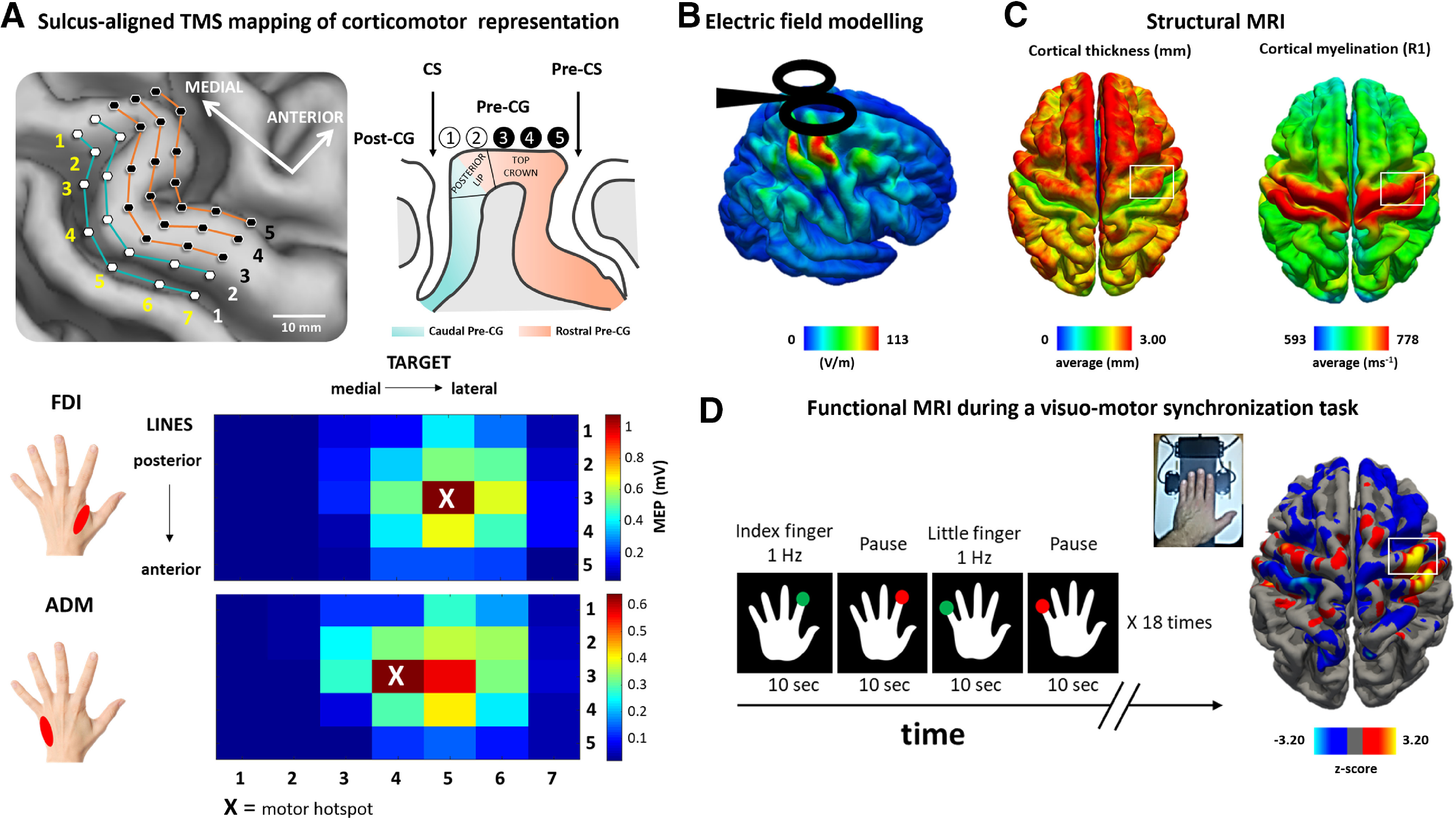
Multimodal mapping of the precentral hand knob. ***A***, Top, Sulcus-aligned mapping with single-pulse TMS. The mapping grid consists of five lines with seven target sites per line. The lines follow the individual shape of the precentral gyrus forming the right precentral hand knob. Single-pulse TMS was applied at each target site, and MEPs were recorded from left FDI and ADM muscles. The first two lines (light blue) corresponded to posterior lip of the (Pre-CG) crown. The remaining three lines (orange) corresponded to the top of the Pre-CG crown till the Pre-CS. Bottom, Color-coded corticomotor maps for the FDI and ADM muscles. Each square represents a stimulation site, and the color codes the mean MEP amplitude. Note that the FDI muscle is represented more laterally respect to the ADM muscle. ***B***, Surface-rendered group map of the simulated electric field strength induced by TMS. ***C***, Structural MRI: average distribution of cortical thickness (top) and cortical myelination (bottom) measured as longitudinal relaxation rate R1 = 1/T1 across all subjects. ***D***, Average fMRI activity for voluntary abduction-adduction movements of the left index finger (FDI) and little finger (ADM) during a visually cued motor task at 1 Hz.

Experimental procedures are illustrated in Figure 1 and comprise corticomotor TMS mapping as well as structural and functional MRI of the whole brain at 3 tesla. All participants underwent structural and functional MRI the day before the TMS mapping experiment. All MRI scans were acquired with a 3 T Verio Scanner and a 32-channel head coil (Siemens).

##### Structural MRI.

Structural T1-weighted images were acquired to assess cortical thickness and to individually identify and track the TMS target points with frameless stereotactic neuronavigation on each participant's macrostructure. The T1-weighted images had an isotropic resolution with a voxel size of 1 mm^3^ (TR = 2300 ms, TE = 2.96 ms, flip angle = 9°). T2-weighted images were acquired to inform offline simulation of the induced electric fields in the precentral gyrus in each individual participant given the intensity of the stimulation and the distance of the coil from the scalp in each condition. T2-weighted whole-brain scans had a voxel size of 1 × 1 × 2 mm^3^ (TR = 10,000 ms, TE = 52 ms, flip angle = 120°). In addition, a whole-brain multiparameter mapping protocol was run to obtain quantitative R1 maps as an index of regional cortical myelination (Helms et al., 2008; Lutti et al., 2010). The protocol is based on multiecho 3D Proton Density- and T1-weighted FLASH (fast low-angle shot) images at 1 mm isotropic resolution, which undergo correction for radiofrequency transmit field inhomogeneities using an EPI (echoplanar imaging)-based B1+ map. The latter is corrected for off-resonance effects using a B0 field map. For further details regarding the sequence parameters, we refer to two publications (Weiskopf and Helms, 2008; Weiskopf et al., 2011).

##### Functional MRI.

Functional MRI used a gradient EPI sequence sensitive to detect task-related blood oxygenation level-dependent (BOLD) changes in tissue contrast (TR = 2.07 s, TE = 30 ms, flip angle = 78°, voxel size = 2 × 2 × 2 mm^3^, axial field of view = 192 × 192 mm). A single brain volume consisted of 25 axial slices covering the upper half of the brain. The axial orientation of the brain volume orientation was slightly tilted backward so that the orientation of the slices was approximately perpendicular to the course of the central sulcus. Three hundred thirty-five brain volumes were acquired during the fMRI session. Two additional short whole-brain EPI scans (62 slices) with the same parameters except for an adjusted TR were recorded for coregistration purposes. Pulse and respiration were recorded with an infrared pulse oximeter and a pneumatic thoracic belt.

Task-related activity changes were mapped with BOLD fMRI while participants performed a repetitive isometric finger abduction task with their left index or little finger (Fig. 1*D*). This task engaged the same muscles that were investigated with TMS, namely the first dorsal interosseous (FDI) muscle and abductor digiti minimi (ADM) muscle. The left hand of the subject was placed on a board fitted with two strain gauge sensors measuring the abduction forces produced with the index or little finger (Fig. 1*D*). The strain gauge sensors were connected to a custom amplifier that converted the measured force to a voltage in the range 0–2.5 V, and this signal was sampled via a USB 2.0 acquisition device (PicoLog 1216, Pico Technology) at a sampling rate of 500 Hz. Involuntary movement of the thumb, the middle finger, and the ring finger were avoided by using adhesive felt. Subjects saw a schematic drawing of the back of the left hand displayed on a video screen that was visible to the subjects via a coil-mounted mirror. Red or green dots were presented every second at the tip of the left index or little finger. Participants had to produce an isometric abduction with the corresponding finger whenever a green dot appeared at the tip of the target finger. Participants had to refrain from any movement, whenever a red dot was presented. Using a block design, the same dot and color were presented 10 times. The duration of presentation of green or red dots within each block was 0.5 s at a constant frequency of 1 Hz without any jitter. The visuomotor synchronization task consisted of blocks of movements (green dots) alternated with blocks without movements (red dots). The four task conditions were always performed in a fixed order and repeated 18 times during the fMRI run (Fig. 1*D*). Stimulus presentation and response recording were controlled by custom-made programs (PsychoPy software, version 1.74.01; www.psychopy.org; Peirce, 2009). Immediately before the fMRI experiment, all participants performed a short training version of the task outside the MRI scanner to get familiarized with the task. We instructed participants to emphasize the timing of movement and try to synchronize as well as possible the movement rate to the pace of the visual cue to the best of their ability. Performance of the subjects in the scanner was controlled by video monitoring. Importantly, we were interested in movement regularity and synchronization to visual inputs, rather than in the accuracy of responding with the correct finger or producing a constant abduction force. Therefore, our task probed visuo-to-motor synchronization of simple repetitive finger movements rather than visuo-to-motor response mapping. We reasoned that the degree of cortical myelination in the precentral hand knob should scale with temporal precision of movement (i.e., the ability to reliably adjust movement repetition rate to the pace of the external cue). Therefore, we hypothesized that high levels of precentral cortical myelination should be associated with low trial-to-trial variability of the between-movement interval during the visually cued movement synchronization task.

##### Transcranial magnetic stimulation.

We applied a sulcus-aligned TMS mapping approach that has been developed by our group (Raffin et al., 2015; Dubbioso et al., 2017; Raffin and Siebner, 2019) to precisely localize the optimal site for evoking MEPs in two intrinsic hand muscles (i.e., precentral motor hotspot) along the rostrocaudal axis in the crown of the precentral gyrus (Fig. 1). Single biphasic TMS pulses were applied over multiple sites overlaying the right precentral hand knob. TMS was performed with a cooled MC-B35 figure-of-eight coil connected to a MagPro X100 stimulation device (MagVenture). We chose a MC-B35 TMS coil, because this coil is small with an average winding diameter of 35 mm to maximize the focality of TMS. Participants were seated in an adjustable armchair with the neck supported by a headrest during TMS mapping. The position of the TMS coil relative to the participant's head was continuously tracked in real time with frameless stereotactic neuronavigation (Localite). Any changes in the TMS position and orientation relative to the scalp were registered and updated online in a 3D space and displayed to the examiner on a screen. Before sulcus-aligned TMS mapping, we located the motor hotspot position of the left FDI muscle using the standard trial-and-error procedure with the handle of the coil angled at 45° relative to the midsagittal line (Groppa et al., 2012). We then determined the resting motor threshold (RMT) of the left FDI muscle, using the maximum-likelihood strategy using parameter estimation by sequential testing approach (Awiszus, 2003).

The TMS-evoked motor responses were recorded with surface electromyography from relaxed left first FDI and ADM using a belly tendon montage (Neuroline 700, Ambu). Trial-wise acquisition of MEPs was controlled by Signal software, and EMG data were stored on a computer for later offline analysis (Cambridge Electronic Design). Surface EMG signals were recorded at a sampling rate of 10 kHz and bandpass filtered (20–3000 Hz) with an eight-channel DC amplifier (1201 micro Mk-II unit, Digitimer, Cambridge Electronic Design).

##### Sulcus-aligned TMS mapping of the motor hand knob.

Standard grid-based TMS mapping of corticomotor representations keeps the coil orientation of the TMS coil identical across all grid sites (Teitti et al., 2008; Diekhoff et al., 2011; Vaalto et al., 2011, 2016; Sarfeld et al., 2012; Ahdab et al., 2014, 2016). This procedure induces electrical tissue currents in the motor hand knob that enter the precentral crown at different angles at the maximally stimulated part of the crown, when placing the coil at different points of the grid. This is problematic because the angle at which the electrical current “hits” the precentral gyrus has a strong impact on the TMS-induced electric field (Thielscher et al., 2011). In addition, many of these mapping studies showed considerable interindividual variation in the hot spot location, with some participants having the hot spot located even in the prefrontal regions (Teitti et al., 2008; Vaalto et al., 2011; Ahdab et al., 2014, 2016).

These considerations prompted us to develop a sulcus-aligned TMS mapping procedure that adjusts the orientation of the TMS coil at each mapping site to the local curvature of the precentral gyrus (Raffin et al., 2015; Dubbioso et al., 2017; Raffin and Siebner, 2019). The procedure exploits the fact that the motor hand representation in the precentral gyrus can be readily identified on structural MRIs by its characteristic knob-like shape (Yousry et al., 1997). The TMS coil is centered on one of seven equidistant target sites placed along the individual gyrus–sulcus border of the hand knob. The coil orientation is adjusted at each target site in a way that TMS always induces the strongest currents in a direction that is perpendicular to the local orientation of the precentral gyrus. This secures the idea that TMS induces the highest electrical field strength in the crown of the precentral hand knob at all stimulation sites.

In this study, we extended our linear sulcus-aligned TMS mapping approach into a two-dimensional TMS mapping procedure to identify interindividual differences in the rostrocaudal peak location of corticomotor excitability in the precentral gyrus. We added four parallel lines rostrally to the central sulcus over the precentral gyrus (Fig. 1*A*). Each of the five parallel lines consisted of seven equidistant targets covering the entire longitudinal extension of the hand knob (35 target sites in total). The distance between two neighboring target sites on the line was 5 mm. The first two lines covered the posterior lip of the precentral crown and the remaining three lines the top (apex) and anterior lip of the precentral gyral crown (Fig. 1*A*). We reasoned that a sulcus-aligned mapping approach would be more sensitive than standard grid-based mapping with a conventional figure-of-eight coil to detect very small interindividual variations of hotspot location. Indeed, personalization to the pre-central sulcus (Pre-CS) shape and a short interline distance rendered our approach more sensitive to subtle shifts in hotspot location in the Pre-CS.

TMS mapping was stereotactically guided using frameless neuronavigation (Localite). First, the head of the subject was coregistered with the individual high-resolution anatomic MRI of the brain via anatomic landmarks (e.g., nasion and crus helicis) by using the surface mapping function of Localite. The root mean square differences between the positions of landmarks in the MRI volume and at the subjects head were ≤2 mm for any TMS session of this study. Furthermore, to verify the quality of the coregistration procedure, we attached small vitamin E capsules (providing a good MRI T1 contrast) to a volunteer's head at different anatomic positions. The software depicted and true positions of the capsules did not show mismatches >1 mm for any position.

The brain surface, derived from the individual T1-weighted MRI, was rendered online, and the sites to be targeted by TMS in the precentral gyrus were marked as dots on the segmented brain of each subject (Fig. 1*A*). Based on anatomic landmarks, a trained investigator (R.D.) manually placed 35 stimulation sites: seven targets for five lines. As in our recent sulcus-aligned mapping studies (Raffin et al., 2015; Dubbioso et al., 2017; Raffin and Siebner, 2019), we chose a biphasic TMS pulse configuration. Biphasic pulse configurations are more efficient to stimulate the M1_HAND_ than monophasic pulses (Lang et al., 2006). This allowed to use of a very focal coil without any heating problems, increasing focality compared with standard coils. The second phase of the biphasic stimulus always produced a current in the precentral gyrus with a caudal-to-rostral (posterior–anterior) direction perpendicularly to the local curvature of the central sulcus (Kammer et al., 2001).

To avoid carryover effects between consecutive stimuli, interstimulus intervals were jittered between 4 and 5 s. For each target, we delivered 20 pulses in two 10-stimuli blocks at an intensity set to 120% of the conventionally defined RMT for left FDI muscle. At this stimulation intensity, we reliably evoked motor responses in the left FDI and ADM muscles. The order of stimulated precentral targets was pseudorandomized across subjects with a fixed target sequence within a subject. The individual coil-positioning parameters were stored by the neuronavigation software for each stimulation position (Localite).

#### Data analysis

##### Motor evoked potentials.

After the experiment, the EMG recordings were visually inspected to remove trials with significant artifacts. The peak-to-peak amplitude of each MEP was extracted trial-by-trial using Signal software (Signal version 4.02 for Windows, Cambridge Electronic Design) in the time window between 10 and 40 ms after the TMS stimulus. For each of the 35 cortical targets, we determined the mean peak-to-peak MEP amplitude and used the mean MEP amplitude to generate muscle excitability maps for the ADM and FDI muscles along the precentral gyrus (Fig. 1*B*). The resulting map indicated the site at which the mean MEP amplitude reached its peak. This “MEP peak” indicates the individual location of the motor hotspot in the precentral hand knob. Please note that we used the motor hotspot location derived from sulcus shape-based TMS mapping for all further analyses. The conventionally identified hotspot location that we had determined by trial-and-error at the start of the experiment to determine RMT was only considered in the analyses involving the simulation of the TMS-induced electrical field strength. In addition, we tested stability and reproducibility of this procedure on a single subject by replicating the TMS mapping procedure 1 week later. We used a custom-made software to extract the Montreal Neurological Institute (MNI) normalized stereotactic *x*-, *y*-, and *z*-coordinates, reflecting the cortical projection of the precentral motor hotspot as revealed by our sulcus-aligned mapping procedure.

We hypothesized that in individuals with a more rostral (anterior) precentral hotspot, TMS elicits premotor cortical excitation more upstream to M1_HAND_ than in individuals with a more posterior (caudal) precentral hotspot. We therefore expected that individuals with a rostral hotspot should also show longer MEP latencies than individuals with a caudal motor hotspot location in the precentral crown. Therefore, we determined the shortest MEP latency for the FDI and ADM muscles for each subject at the motor hotspot location. The shortest latencies were identified and measured by visual inspection of superimposed MEP waveforms (Chen et al., 2008; Groppa et al., 2012). This measurement was performed by an experienced neurophysiologist (P. J. Sørensen, DRCMR, Copenhagen University) who was blinded with respect to the protocol setup. We opted for corticomotor latency, because this measure had been used in previous studies (Hamada et al., 2013; Volz et al., 2015) that successfully explore intraindividual effects of changing current direction on corticomotor conduction time, but acknowledge that the estimation of corticomotor conduction time would have been preferable to minimize the contribution of peripheral motor conduction time (Groppa et al., 2012).

##### Cortical thickness, folding, and myelination.

Cortical reconstruction was performed with the FreeSurfer image analysis suite, version 6.0.0 (http://surfer.nmr.mgh.harvard.edu/; Fischl and Dale, 2000). Using this approach, the gray and white matter surfaces were defined by an automated brain segmentation process. If required, an experienced investigator manually corrected the automated segmentation, following the procedures outlined at https://surfer.nmr.mgh.harvard.edu/fswiki/Edits. The processes of surface extraction and inflation generated a number of well known feature descriptors for the geometry of the cortical surface. These included the following: surface curvature estimated from the mean curvature (or average of the principal curvatures) of the white matter surface (Pienaar et al., 2008); and cortical thickness estimated at each point across the cortex by calculating the distance between the gray/white matter boundary and the cortical surface. Individual whole-brain surface maps were smoothed with a 5 mm 2D Gaussian smoothing kernel (Fischl and Dale, 2000), and the effect of surface curvature on cortical thickness was regressed out (Glasser et al., 2016). Using the FreeSurfer spherical registration method, the individual curvature-corrected cortical thickness maps were registered to a common FreeSurfer template surface (fsaverage) for visualization and group analysis (Fig. 1*C*; Fischl, 2012).

The data of the multiparameter mapping protocol was processed using a Voxel-Based Quantification (VBQ) toolbox developed for Statistical Parametric Mapping version 8 (SPM8; www.fil.ion.ucl.ac.uk/spm/).

Because curvature-associated modulations of myelination can obscure or distort myelination changes because of other variations in cytoarchitectonics and myeloarchitectonics (Annese et al., 2004), we have regressed out curvature and used for analysis the curvature-corrected R1 value variations, smoothed with a 5 mm 2D Gaussian smoothing kernel (Fischl and Dale, 2000). The individual maps were registered onto the FreeSurfer group template for visualization and group analysis (Fig. 1*C*).

We were primarily interested in estimating regional cortical myelination as reflected by R1 values derived from quantitative structural MRI in the right precentral gyrus forming the motor hand knob. Therefore, we defined the right motor hand knob as precentral region of interest (ROI), covering the M1_HAND_ and the adjacent PMd directly anterior to it. Within this ROI, we separately defined the caudal pre-central gyrus (Pre-CG), namely the gyral wall facing the central sulcus and the rostral Pre-CG, the gyral crown, and gyral wall facing pre-central sulcus. The border between the caudal and the rostral Pre-CG was manually delineated on the average group image generated in FreeSurfer by using a line perpendicular to the cortical surface originating at the maximal convexity of the posterior lip region.

**Figure 2.**
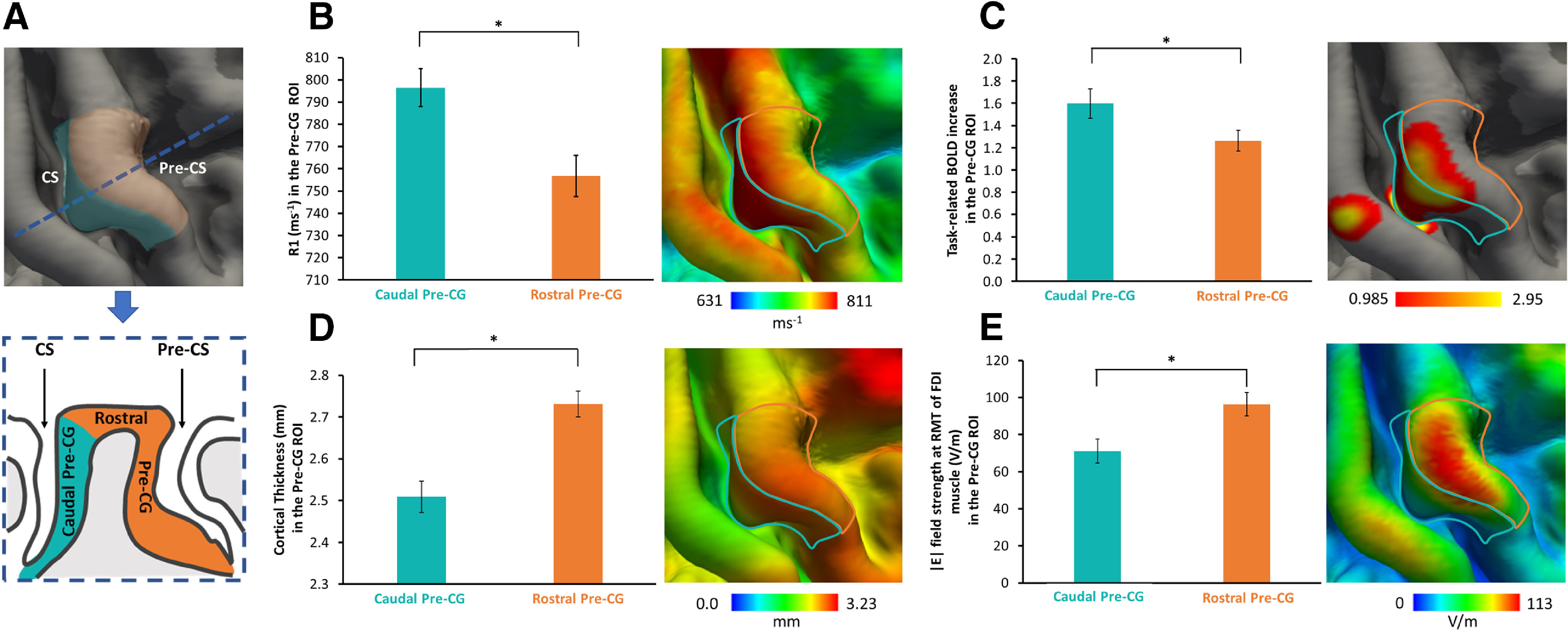
Structural, functional, and electrical field properties of the right Pre-CG. ***A***, Left, Right precentral ROI considered for the analyses, namely the light blue mask including the gyral wall facing the Central Sulcus (CS), caudal Pre-CG ROI, and the orange mask composed of the gyral crown and gyral wall facing the Pre-CS, rostral Pre-CG ROI. Bottom, Cross section (following the blue dotted line) of Pre-CG with a schematic representation of caudal Pre-CG and rostral Pre-CG ROIs. ***B–E***, Significant differences are evident between the caudal Pre-CG and rostral Pre-CG ROIs regarding the cortical myelin content (***B***), functional activation during the visually cued movement repetition task (***C***), mean cortical thickness (***D***), and mean TMS-induced field strength (***E***). Each panel consists of a bar plot representing the regional mean (left) and a surface-rendered voxel-wise group map, including the borders of the two ROIs. *p < 0.01; paired *t*-test.

Then, the mediolateral and anteroposterior borders of the overall ROI were defined based on the grid we used for precentral TMS mapping to achieve a reliable comparison of the MRI and TMS data. First, we considered the border of the grid by taking the grand mean of MNI normalized stereotactic *x*-, *y*-, and *z*-coordinates of the stimulation points forming the grid border of all participants. These coordinates were then projected on the flat FreeSurfer template surface (fsaverage) by using a custom-made MATLAB script. Each point was then connected to form a rectangle with a size of ∼2 × 3 cm, using the function tksurfer_labeledit implemented in the FreeSurfer software package. The rectangle was finally projected onto the pial surface (Fig. 2*A*). Individual mean cortical thickness values and R1-based cortical myelination estimates were extracted from the two precentral ROIs and used for correlational analyses.

##### Task-related fMRI and behavioral data.

We used the SPM8 (Wellcome Department of Imaging Neuroscience, UCL; http://www.fil.ion.ucl.ac.uk) for preprocessing and statistical analysis of the functional MRI data. The first four volumes of a session (dummy images) were discarded from further analysis. Whole-brain EPIs, including reversed-phase EPI, were recorded to facilitate the accurate registration of the EPI data to the individual T1-weighted image. The EPI time series was motion corrected, brain extracted, and smoothed with a 1 mm two-dimensional Gaussian smoothing kernel (Fischl and Dale, 2000). We chose a small smoothing kernel to minimize the smearing of task-related somatosensory processing in the post-central gyrus into the pre-central gyrus.

We specified a first-level general linear model to assess differences in brain activity between the movement and rest blocks for each hand muscle. Two regressors-of-interest were defined for the blocks requiring isometric abduction movements of the left index finger (engaging the FDI muscle) or little finger (engaging the ADM muscle). To account for shifts in the onset of the hemodynamic response, temporal derivatives of the resulting time courses, motion, respiration, and cardiac cycle were included in the model as regressors-of-no-interest (Friston et al., 1997; Smith et al., 2004). After model estimation, *z*-statistic images were calculated for the resulting maps of the parameter estimates, and a corrected statistical threshold of *p* < 0.05 was applied at the cluster level based on Gaussian random field theory (Worsley et al., 1996). The cluster extent threshold was set to an uncorrected *p* < 0.001 (here corresponding to a *z* score of 3.2). For reporting, the *z*-statistic images were projected into MNI space based on a nonlinear registration of the T1-weighted structural MRI on the MNI152 template (using FSL FNIRT; http://fsl.fmrib.ox.ac.uk/fsl/slwiki). In addition, average activation maps across subjects were rendered on the FreeSurfer group template (Fig. 1*D*) for visualization using the registration procedures as described in the online FreeSurfer tutorial (https://surfer.nmr.mgh.harvard.edu/fswiki/FsTutorial/FslFeatFreeSurfer).

During the visually cued isometric finger abduction task, we extracted the mean interval between two consecutive isometric contractions (i.e., intermovement interval), its SD. Specifically, the signal was thresholded at a level of 0.6 V and the findpeaks MATLAB function was used to identify peaks with a minimum distance of 400 ms and a peak prominence of one-third of the maximum force value exerted by the subject. As the movement onsets were quite steep, we found it reasonable to use these peaks to define movement intervals. Importantly, the limited dynamic range of the force measurement setup caused the exerted force to often go beyond the maximum, which meant that the force measurements were only of limited practical use in this setting. Last, we calculated the coefficient of variation (CV) by dividing the SD with the mean to quantify between-trial dispersion of movement timing. The CV of the intermovement interval indicates how well participants reproduced the one-per-second interval as signaled by the visual cue.

##### Electric field simulations.

For each participant, we performed simulations of the electric fields that were generated in the right precentral gyrus by the TMS pulse using SimNIBS software 2.0 (www.simnibs.org). A realistic head model was automatically generated for each participant from the individual T1-weighted and T2-weighted MR images as described previously (Thielscher et al., 2015; Bungert et al., 2017; Weise et al., 2020). Electric field simulations were calculated for the coil position which elicited the highest mean MEP amplitude (i.e., the individual precentral motor hotspot) in left FDI muscle and a stimulation intensity scaled to the individual RMT of FDI muscle to clearly visualize the effect of gyral anatomy on TMS-induced field strength. The TMS-related parameters to compute the E-field strength (i.e., coil location, stimulus intensity) were obtained before the main TMS mapping experiment, when we conventionally determined the individual RMT of the FDI muscle by trial-and-error (Groppa et al., 2012). Hence, the E-field calculations represent the maps that one normally would derive in standard TMS experiments at the individual precentral motor hot spot. The vector potential of the MC-B35 coil was precalculated using a coil model consisting of a superposition of 1248 magnetic dipoles, as described in the study by Thielscher and Kammer (2004). To obtain average electric fields across subjects, the electric fields were interpolated in the middle of the segmented cortical gray matter and transformed to the FSAverage template (Fischl, 2012) for second-level group analyses. We then created a group map of the electrical field distributions for the motor hotspot locations and statistically compared the TMS-induced electrical field distributions in the right precentral hand knob between the M1_HAND_ and PMd group. Since the rostral M1_HAND_ is confined to the posterior lip region of the precentral gyrus (Geyer et al., 1996), we hypothesized that the M1_HAND_ group should display a higher electrical field magnitude in the posterior lip region relative to the PMd group.

#### Statistical group analyses

All of the statistical computations were performed using SPSS Statistics software (version 22 for Windows, IBM). Before applying parametric statistical tests, the normal distribution of all variables was verified by means of a Kolmogorov–Smirnov test.

In a first set of analyses, we explored the structural, functional, and |E| field properties of the rostral and caudal part of the precentral motor hand knob. Individual estimates of myelination (derived from R1 mapping), cortical thickness (derived from T1-weighted MRI scans), mean electric field strengths, and mean task-related BOLD signal increase for index, and little finger movements were extracted from the two precentral ROIs in the right precentral hand knob, corresponding to the caudal Pre-CG and rostral Pre-CG. For each variable, we computed separate paired *t* tests to assess morphofunctional differences between the two precentral ROIs. Finger movement (index vs little finger) was included as additional within-subject factor in the ANOVA models analyzing fMRI data and related performance measures.

A second set of analyses focused on the neurophysiological data recorded during sulcus shape-based TMS mapping of the left FDI and ADM muscles. Our primary interest was to capture interindividual differences in corticomuscular representation in the right precentral motor hand knob along the anterior–posterior axis. To quantify interindividual variation in rostrocaudal corticomuscular representation, we derived a composite measure for each participant that integrated the spatial (*y*-coordinate of precentral motor hot spot) and the temporal dimension (corticomotor MEP latency at motor hot spot) of hot spot rostrality. We reasoned that the most robust way of quantifying functional rostrality of the corticomotor representation would be to derive a composite measure that integrates both the spatial and temporal dimension of functional rostrality. The spatial dimension specifies how far the primary site of stimulation is away from the corticospinal output neurons, and the temporal dimension reflects how long it takes from the primary side to induce a transsynaptic excitation of the corticospinal output neurons.

In each participant, we first took the shortest MEP latency that had been obtained at the motor hotspot for both ADM and FDI muscles, and the corresponding *y*-coordinate of the respective muscle. We normalized each measure by scaling it between 0 and 1, with 0 corresponding to the shortest latency and the most posterior hotspot and 1 to the longest latency and most anterior hotspot. The two normalized variables were then multiplied yielding a muscle- and subject-specific hotspot rostrality index. To test whether the spatial and temporal dimensions of the hotspot rostrality index are related, we calculated the Pearson's correlation coefficient between the individual *y*-coordinate of the hot spot and the shortest MEP latency at the motor hotspot for the respective muscle.

The third set of analyses addressed the main question of this study and tested for function–structure relationships in the right precentral motor hand knob. We hypothesized that interindividual variations in precentral myelin content would account for between-subject differences in precentral motor function (i.e., hot spot rostrality and task-related activation) and task performance (i.e., temporal synchronization of repetitive finger movement). Using the mean R1 signal of the entire Pre-CG ROI as an index of cortical myelination, we calculated the Pearson's correlation coefficient between the mean precentral R1 value and individual functional readouts such as the spatiotemporal rostrality index of the precentral motor hot spot, the mean task-related activation, and the stability of the between-movement interval during the visually cued movement synchronization task. Correlation analyses were conducted for each muscle (motor hotspot rostrality) or finger (task-related activation and performance during the cued movement synchronization task). The significance threshold was set at *p* < 0.05, and the Bonferroni procedure was used to correct for multiple comparisons performed in each set of analyses. Data are given as the mean (±SEM) if not otherwise specified.

We computed several follow-up analyses. We repeated the correlational analyses using only the mean MRI signal from the rostral and caudal Pre-CG ROI to see whether linear structure–function relationships were evenly expressed in the two ROIs. We also performed the same set of analyses using mean cortical thickness rather than the mean R1 signal to exclude that the results were driven by differences in the thickness of the precentral hand knob. We also computed exploratory correlational analyses to assess the relationship between spatiotemporal hotspot rostrality of the FDI and ADM hot spots and functional MRI-based and behavioral readouts.

To visualize the spatial expression of significant correlations at the voxel level within the right precentral hand knob, we computed additional surface-based analyses within the precentral ROI on the FsAverage template by using FreeSurfer software. These analyses were performed vertex-wise, followed by cluster-wise corrections for multiple comparisons based on the method suggested by Hagler et al. (2006; cluster-determining threshold, *p* < 0.001; cluster-wise correction, *p* < 0.05). Age at the time of MRI and sex were included in the model as nuisance variable. Finally, a separate surface between group analysis was used to compare the spatial distribution difference of E-field between the two groups of participants (posterior lip stimulation vs top of crown stimulation). Since we had a highly specific anatomic hypothesis (posterior lip region), the explorative between-group analysis of electrical field distribution in precentral gyrus applied a more liberal cluster extent threshold of *p* < 0.01.

## Results

### Cortical myelination, thickness, and curvature

The structural properties of the right precentral hand knob were assessed with quantitative structural MRI, using the mean R1 value of the caudal and rostral parts of the Pre-CG as an index of regional myelination (for details, see Materials and Methods). The R1 value was available in only 20 of the 24 subjects, as the quantitative MRI data of four subjects had to be excluded because of head movement-related artifacts. The caudal part of the Pre-CG showed higher mean R1 values than its rostral part (caudal Pre-CG ROI, 796.48 ± 8.51 ms^−1^; vs rostral Pre-CG ROI, 756.75 ± 9.29 ms^−1^; paired *t* test: *t*_(19)_ = 5.069, *p* < 0.001; Fig. 2*B*). We also ran voxel-wise analysis and surface rendering to visualize the regional expression of R1 values, which showed that the regional R1 signal gradually decreased along a caudal-to-rostral gradient in the precentral hand knob (Fig. 2*B*). To capture macrostructural features of the precentral motor hand knob, we derived cortical thickness and curvature from the T1-weighted MRI images. On average, the cortex was thicker in the rostral part of the precentral motor hand knob than in its caudal part (caudal Pre-CG ROI, 2.51 ± 0.04 mm; vs rostral Pre-CG ROI, 2.73 ± 0.03 mm; paired *t* test: *t*_(23)_ = −6.431, *p* < 0.001; Fig. 2*D*). Cortical folding, indexed by regional mean curvature was larger in the caudal part than in the rostral part of the precentral motor hand knob (caudal Pre-CG ROI, 0.02 ± 0.01 mm^−1^; vs rostral Pre-CG ROI, −0.08 ± 0.01 mm^−1^; paired *t* test: *t*_(23)_ = 42.368, *p* < 0.001).

### Precentral corticomotor representations

**Figure 3.**
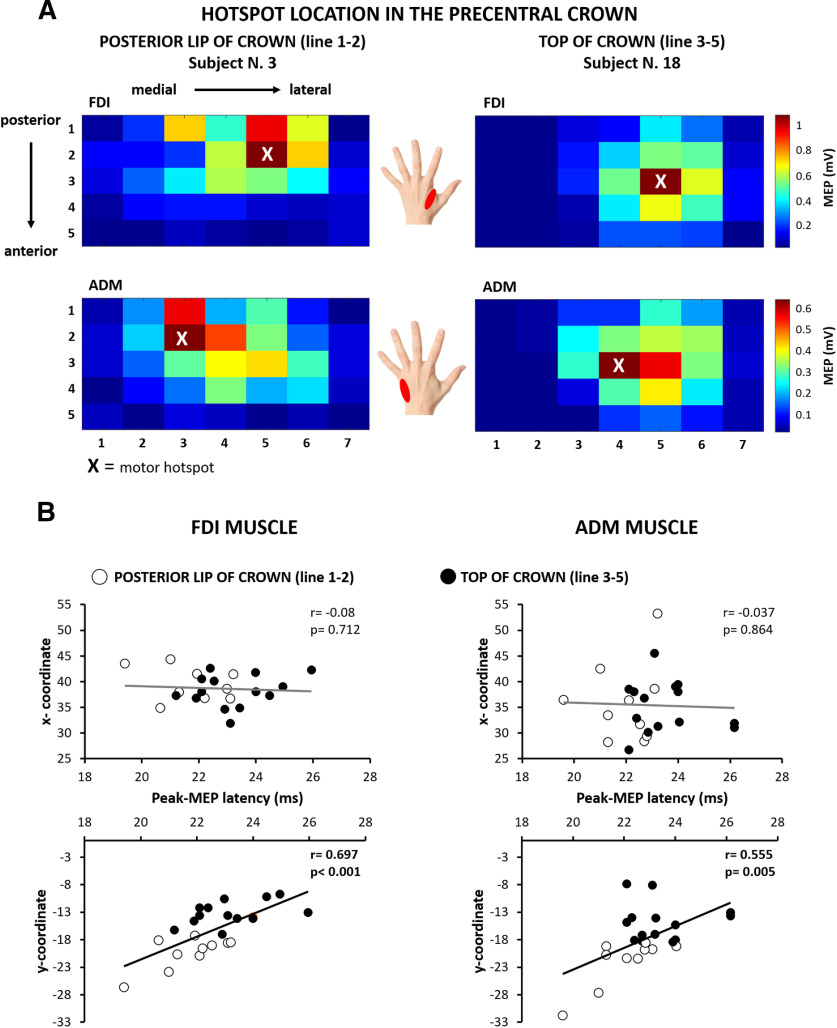
Corticomotor maps of the right precentral hand knob derived from sulcus shape-based TMS mapping. ***A***, Two-dimensional color-coded map illustrating the spatially distribution of corticomotor excitability in the right precentral hand knob. The motor hotspot is indicated by an “X.” Left, Map of an individual (subject 3) with caudal hotspot location in the posterior lip of the precentral gyrus for the FDI muscle (top) and ADM muscle (bottom). Right, Map of an individual (subject 18) with a more rostral hot spot at the top of the precentral crown. Each square corresponds to a specific target site determined by its mediolateral (targets 1–7) and posterior–anterior (lines 1–5) positions. ***B***, Scatter plots plotting the *x*-coordinate (mediolateral direction) or *y*-coordinate (posterior–anterior direction) coordinates of the motor hot spot in MNI space against the shortest MEP latency recorded at the motor hot spot location. Significant correlations with MEP latencies were only found for the posterior–anterior position of the hotspot coordinates (*y*-coordinates) but not for the mediolateral position (*x*-coordinates). Open black circles indicate participants with a more caudal hot spot location in the posterior lip of Pre-CG (located on lines 1 and 2 of the mapping grid). Close black circles indicate participants with a more rostral hot spot location at the top of the Pre-CG crown (located on line 3 or 4 of the mapping grid). Please, note that the labeling of the *x*-axes is identical for all four scatter plots.

In each participant, the mean peak-to-peak MEP amplitude at each cortical stimulation site was used to create two-dimensional maps of the corticomotor representations of the FDI and ADM muscles (Fig. 3*A*). This enabled us to identify the precentral motor hotspot for each muscle (i.e., the stimulation site at which mean MEP amplitude was the largest). The mean MEP amplitude at the motor hotspot was 1.11 mV (±0.17 mV) for the FDI muscle and 0.50 mV (±0.08 mV) for the ADM muscle, reflecting the fact that MEP amplitudes were overall larger for the FDI muscle. Since we mapped the spatial representation of MEP amplitudes along five parallel lines in parallel to the curvature of the precentral gyrus, we were able to estimate the “rostrality” of the individual hotspot along the anterior–posterior dimension of the precentral gyrus. In agreement with our hypothesis, the position of individual motor hotpots along the rostrocaudal direction varied across participants. Based on the rostrality of precentral hotspot location, we assigned participants to a “rostral hotspot” group in which the hotspot located on one of the three anterior lines (*n* = 14) or to a “caudal hotspot” group in which the hotspot located on one of the two posterior lines close to the central sulcus (*n* = 10). Table 1 summarizes mean group data for the entire group as well as for the rostral and caudal hotspot subgroups, separately. Groups were matched in terms of overall efficacy to excite the corticomotor output (Table 1). Mean MEP amplitudes at hotspot and cortical excitation thresholds for evoking a motor response did not differ between the rostral and caudal hotspot subgroups for both muscles (FDI muscle: *t*_(22)_ = 14.461, *p* = 0.434; ADM muscle: *t*_(22)_ = 21.611, *p* = 0.503; unpaired *t* test).

**Table 1.** The table lists demographic and electrophysiological data (mean ± SEM)

	All participants (*n* = 24)	Caudal hotspot group (*n* = 10)	Rostral hotspot group (*n* = 14)	Statistic, *p* value
Sex (male/female)	12/12	5/5	7/7	χ^2^ = 0, *p* = 1
Age (years)	24.2 ± 0.9	22.6 ± 1.1	25.4 ± 1.3	*t*_(22)_ = −1.699, *p* = 0.11
Height (cm)	172.9 ± 6.0	171.5 ± 4.7	173.9 ± 6.8	*t*_(22)_ = −1.004, *p* = 0.326
RMT of FDI muscle (%MSO)	58.1 ± 2.2	55.3 ± 2.4	60.1 ± 3.4	*t*_(21.6)_ = −1.144, *p* = 0.27
MEP latency at hotspot (ms)				
Left FDI muscle	22.6 ± 0.3	21.7 ± 0.4	23.3 ± 0.4	*t*_(20.9)_ = −2.952, *p* = 0.008
Left ADM muscle	22.9 ± 0.3	22.0 ± 0.4	23.5 ± 0.4	*t*_(21.1)_ = −3.033, *p* = 0.006
MEP amplitude at hotspot (mV)				
Left FDI muscle	1.1 ± 0.2	1.3 ± 0.3	1.0 ± 0.2	*t*_(14.46)_ = 0.804, *p* = 0.43
Left ADM muscle	0.5 ± 0.1	0.4 ± 0.1	0.6 ± 0.1	*t*_(21.6)_ = −0.682, *p* = 0.5
MNI coordinates of motor hotspot				
Left FDI muscle (*x*-coordinate)	38.7 ± 0.6	39.4 ± 1.0	38.1 ± 0.8	*t*_(19.1)_ = 1.02, *p* = 0.32
Left FDI muscle (*y*-coordinate)	−16.6 ± 1.0	−21.3 ± 1.2	−13.2 ± 0.6	*t*_(13)_ = −6.11, *p* < 0.001
Left FDI muscle (*z*-coordinate)	66.5 ± 0.6	65.2 ± 0.9	67.4 ± 0.7	*t*_(16)_ = −1.873, *p* = 0.08
Left ADM muscle (*x*-coordinate)	35.4 ± 1.2	35.8 ± 2.4	35.1 ± 1.3	*t*_(14.35)_ = 0.269, *p* = 0.79
Left ADM muscle (*y*-coordinate)	−17.8 ± 1.1	−21.7 ± 1.4	−15.0 ± 1.0	*t*_(16.7)_ = −3.85, *p* = 0.001
Left ADM muscle (*z*-coordinate)	69.1 ± 1.0	67.8 ± 1.6	70.1 ± 1.3	*t*_(18.8)_ = −1.113, *p* = 0.28
Spatiotemporal rostrality index				
Left FDI muscle	0.34 ± 0.05	0.14 ± 0.03	0.49 ± 0.05	*t*_(15.6)_ = −6.568, *p* < 0.001
Left ADM muscle	0.32 ± 0.04	0.18 ± 0.03	0.42 ± 0.05	*t*_(16.7)_ = −3.850, *p* < 0.001

%MSO, percentage of maximum stimulation output. The *p* values refer to between-group comparisons of the mean values of variables (caudal hotspot group vs rostral hotspot group).

We extracted the stereotactic coordinates to systematically assess the topographical distribution of the motor hotspots in the precentral motor hand knob. Using the stereotactic hotspot coordinates as a dependent variable, we computed a mixed-model ANOVA using group assignment as a between-subject factor, and hand muscle (FDI muscle vs ADM muscle) and the axis of stereotactic coordinates (*x*-, *y*-, and *z*-direction) as within-subject factors.

The ANOVA validated our group assignment, showing an interaction between coordinates and group (*F*_(2,44)_ = 6.049, *p* = 0.005). *Post hoc* analyses were performed to test for between-group differences of hotspot locations along the *x*-, *y*-, and *z*-directions. The rostral hotspot and caudal hotspot groups only differed with respect to their *y*-coordinates, corresponding to the sagittal (anterior–posterior) dimension in stereotactic space. The mean *y*-coordinates of both muscles were −21.5 ± 1.3 in the rostral hotspot group and −14.1 ± 0.7 in the caudal hotspot group (*t*_(22)_ = −7.382; *p* < 0.001; Bonferroni-corrected *t* test). The ANOVA also showed an interaction between coordinates and muscle (*F*_(2,44)_ = 8.299, *p* = 0.001), confirming a significant difference in precentral location between the FDI and ADM motor hotspots. Specifically, ADM muscle was located more medially (*t*_(22)_ = 3.312; *p* = 0.005; Bonferroni-corrected *t* test) and superiorly (*t*_(22)_ = −2.598; *p* = 0.005; Bonferroni-corrected *t* test) relative to the hotspot of the FDI muscle. This finding replicates our previous sulcus-aligned mapping studies, using a single line of targets placed at the caudal border of the precentral crown (Raffin et al., 2015; Dubbioso et al., 2017; Raffin and Siebner, 2019). Finally, ANOVA also revealed main effects of the factors group (*F*_(1,22)_ = 47.491, *p* < 0.001) and coordinates (*F*_(2,44)_ = 251.644, *p* < 0.001).

### Spatiotemporal “rostrality” of precentral motor hotspot

We hypothesized that the “spatial rostrality” of the motor hotspot scaled linearly with its “temporal rostrality,” resulting in longer cortico-to-motor conduction time. We therefore tested for a positive linear relation between the anteroposterior coordinate (*y*) of the motor hotspot and the shortest MEP latency evoked at the hotspot. We found that individual corticomotor latencies scaled positively with the spatial rostrality of individual motor hotspot locations in the precentral hand knob (Fig. 3*B*). The more anterior (rostral) the motor hotspot was located along the anterior–posterior (sagittal) direction, the longer was the corticomotor latency. Considering the data of all participants, we found a significant positive linear relationship between *y*-coordinate of the motor hotspot and shortest MEP latency at the hotspot, both for the FDI muscle (*r* = 0.697, *p* < 0.001) and ADM muscle (*r* = 0.555, *p* = 0.005). No such relationship was found for the mediolateral *x*-coordinate (Fig. 3*B*) and superior–inferior *z*-coordinate (FDI muscle: *r* = 0.084, *p* = 0.697; ADM muscle: *r* = 0.109, *p* = 0.614). This association confirms our hypothesis that cortical precentral excitation occurs functionally more “upstream” to the cortico-motoneuronal neurons, when the preferential target site is located more rostrally in the crown of the precentral hand knob.

Our sulcus-aligned TMS mapping procedure yielded a spatial (*y*-coordinate) and a temporal (MEP latency) rostrality measure of the individual TMS target site in the gyral crown of the precentral hand knob. Considering both the spatial and temporal rostrality dimensions, we computed a “spatiotemporal rostrality index” of the TMS hotspot (for details, see Materials and Methods). This spatiotemporal rostrality index reflects how much TMS preferentially excites cortical neurons in the caudal PMd or M1_HAND_. At the individual level, the spatiotemporal rostrality index of the FDI and ADM muscles showed a positive linear relationship (*r* = 0.819, *p* < 0.001), showing that the hotspot rostrality of the two hand muscles was consistently expressed at the single-subject level.

### Precentral myelination scales with spatiotemporal rostrality of precentral motor hotspot

**Figure 4.**
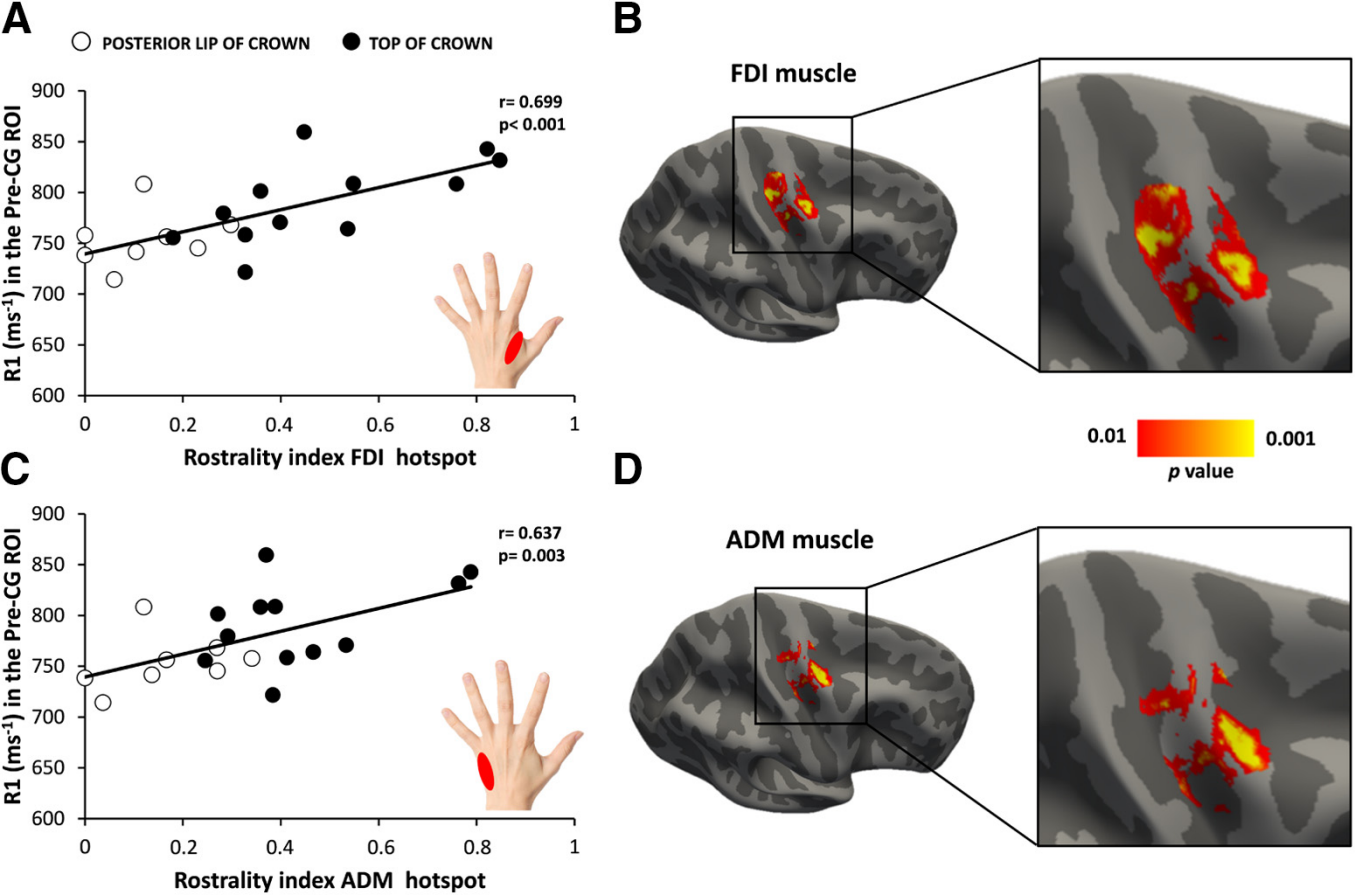
A higher cortical myelin content in right precentral motor hand knob is associated with a more rostral hot spot location. ***A***, ***C***, Positive linear relationship between cortical myelination (as indexed by the mean R1 signal) in the precentral hand knob and the spatiotemporal rostrality index of the TMS hot spot for the FDI muscle (***A***) and ADM muscle (***C***). Open and closed black circles indicate subjects with hotspots located in the posterior lip of precentral crown and top of the precentral crown, respectively. ***B***, ***D***, Surface-rendered statistical parametric maps: the maps show voxels with a significant positive relationship between the precentral myelin-related signal and rostrality index for FDI (***B***) and ADM (***D***) hot spots. Statistical maps are thresholded at *p* uncorrected < 0.01 for illustrative purposes.

The regional myelination of the precentral hand knob as indexed by the mean R1 value showed a significant positive linear relationship with individual spatiotemporal rostrality index. The higher the mean R1 signal in the right precentral hand knob, the higher was the rostrality index of the precentral motor hot spot (Fig. 4). This positive relationship was found for the FDI hotspot (*r* = 0.699, *p* < 0.001; Fig. 4*A*) and ADM hotspot (*r* = 0.637, *p* = 0.003; Fig. 4*C*). Surface-based correlation analyses pinpointed a rostrolateral cluster in the anterior lip region of the precentral crown where regional R1 values correlated most strongly with the individual spatiotemporal rostrality index with peak correlation at *x* = 32.6, *y* = −11.3, *z* = 53.6 for the FDI muscle (Fig. 4*B*) and at *x* = 34.8, *y* = −11.6, *z* = 59.2 for ADM muscle (Fig. 4*D*). A second cluster was located dorsal and medially in the posterior lip region of the precentral gyrus. Correlation peaked at *x* = 26.6, *y* = −22.7, *z* = 51.8 for the FDI muscle and at *x* = 29.3, *y* = −21.8, *z* = 58.7 for the ADM muscle. Both hand muscles expressed a positive linear relationship in the caudal Pre-CG (FDI muscle: *r* = 0.720, *p* < 0.001; ADM muscle: *r* = 0.591, *p* = 0.006) and rostral Pre-CG (FDI muscle: *r* = 0.625, *p* = 0.003; ADM muscle: *r* = 0.571, *p* = 0.007).

At the individual level, regional thickness of the right precentral gyrus did not predict spatiotemporal rostrality of the precentral motor hotspot. No significant correlation was found between cortical thickness and the individual rostrality index for both muscles (FDI muscle: *r* = −0.230, *p* = 0.280; ADM muscle: *r* = −0.277, *p* = 0.190). There was also no significant correlation between the mean curvature of the right precentral gyrus and the rostrality index for both muscles (FDI muscle: *r* = 0.146, *p* = 0.495; ADM muscle: *r* = −0.021, *p* = 0.924). Together, these negative results show that the association between regional myelination and spatiotemporal rostrality of the precentral hotspot was not driven by differences in cortical volume and mean curvature.

### Myelination of the precentral hand knob has functional and behavioral correlates

**Figure 5.**
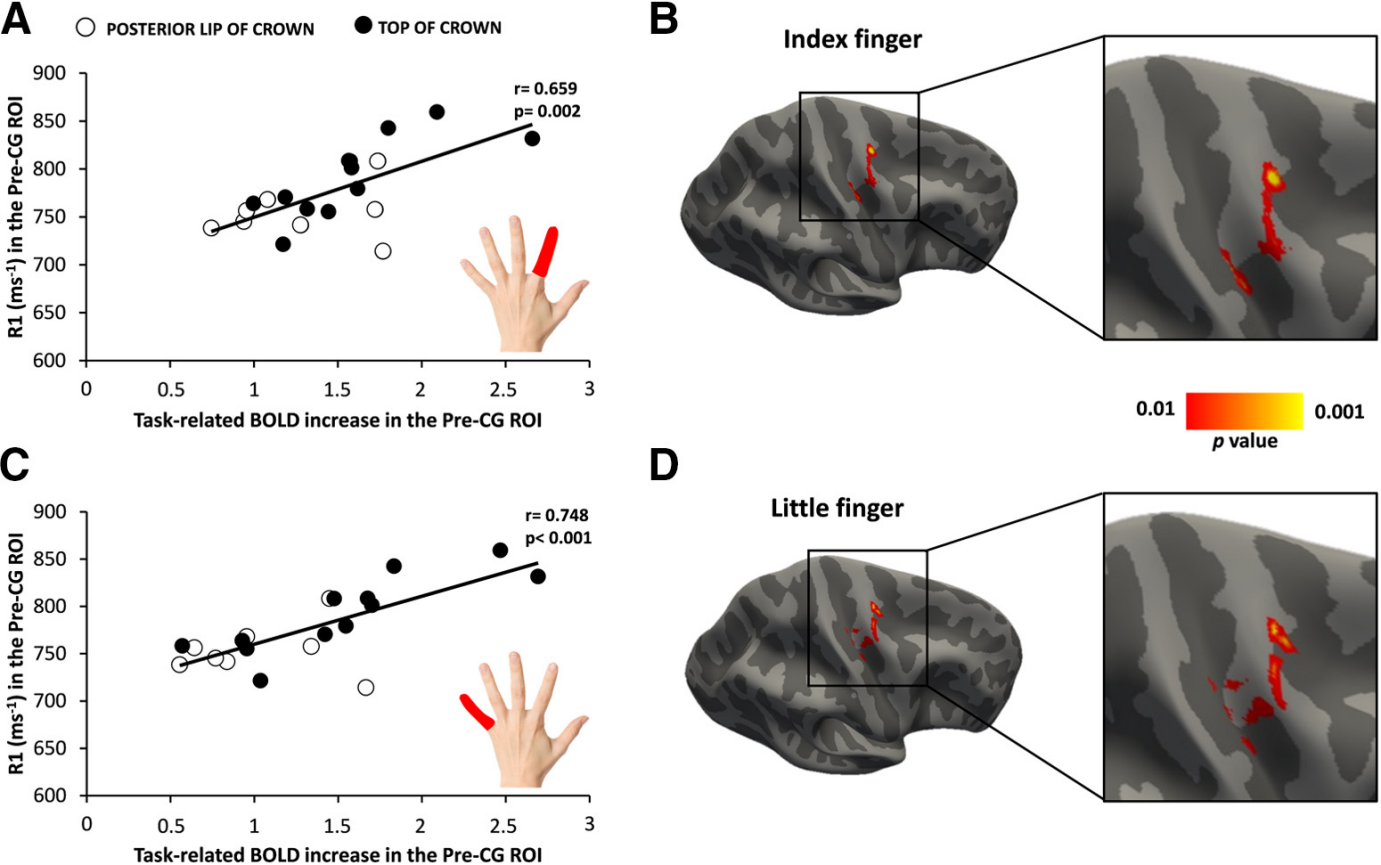
A higher cortical myelin content in right precentral motor hand knob is associated with a higher functional activation during visually cued repetitive finger movements. Positive linear relationship between cortical myelination (as indexed by the mean R1 signal) in the precentral hand knob and the BOLD signal increase during the visuomotor abduction task. ***A***, ***C***, The same positive relationship became evident when the task was performed with the left index finger (***A***) or little finger (***C***). Open and closed black circles indicate subjects with hotspots located in the posterior lip of precentral crown and the top of the precentral crown, respectively. ***B***, ***D***, Surface-rendered statistical parametric maps: the maps show voxels with a significant positive relationship between the precentral myelin-related signal and the task-related BOLD increase for index finger (***B***) and little finger (***D***). Statistical maps are thresholded at *p* uncorrected < 0.01 for illustrative purposes.

Our multimodal mapping approach revealed a link between cortical myelin content and functional corticomotor representations in the precentral motor hand knob and timing proficiency during stereotyped finger movements. While the right precentral motor hand knob was consistently activated when participants performed visually cued finger movements at a repetition rate of 1 Hz, the rostral part of the precentral mean task-related BOLD increase was stronger in the caudal Pre-CG ROI (1.6 ± 0.13) compared with the rostral Pre-CG ROI (1.26 ± 0.09; paired *t* test: *t*_(23)_ = 3.401, *p* = 0.002; Fig. 2*C*). We tested whether the degree of precentral myelination predicts the magnitude of task-related regional activation and task performance. We found a positive linear correlation between the mean cortical R1 signal within the precentral motor hand knob and the magnitude of cortical activation during the visuomotor synchronization task (Fig. 5). This positive relationship was found regardless of whether the task was conducted with the index finger (*r* = 0.659, *p* = 0.002; Fig. 5*A*) or little finger (*r* = 0.748, *p* < 0.001; Fig. 5*C*). Surface-based correlation analyses located the regional expression of this relationship to a rostrolateral cluster in the anterior lip region of the precentral crown where regional R1 values correlated most strongly with regional task-related activation during repetitive movements with the index finger (peak correlation at *x* = 28.3, *y* = −14.4, *z* = 63.1; Fig. 5*B*) or little finger (*x* = 27.6, *y* = −14.1, *z* = 65.2; Fig. 5*D*). The linear relationship between cortical myelination and task-related activation was expressed in the rostral precentral ROI (FDI muscle: *r* = 0.655, *p* = 0.002; ADM muscle: *r* = 0.554, *p* = 0.011), but also in the caudal precentral ROI for the ADM muscle (*r* = 0.591, *p* = 0.006) and trend-wise for the FDI muscle (*r* = 0.404, *p* = 0.078).

**Figure 6.**
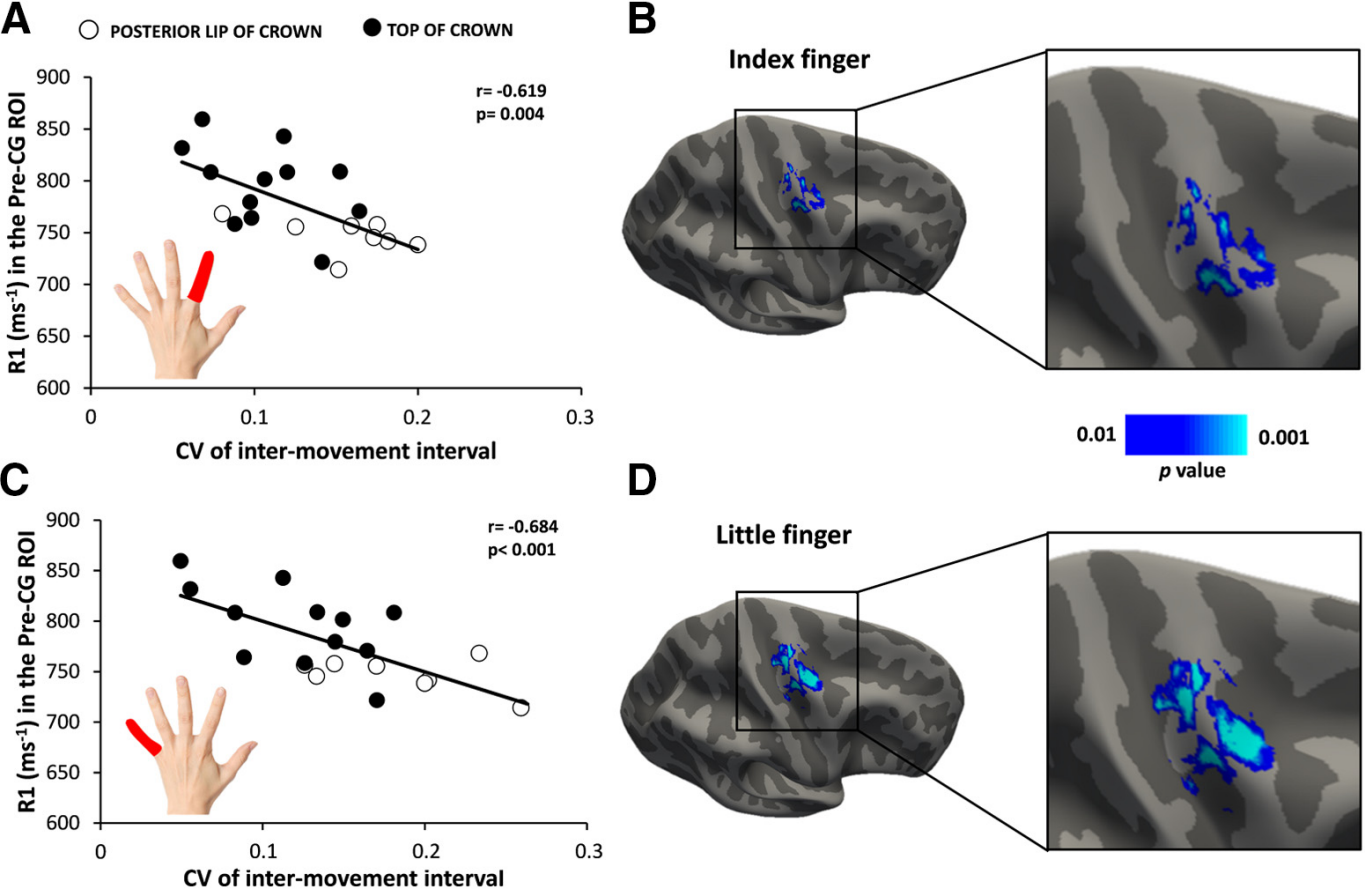
Relationship between the cortical myelin content in right precentral motor hand knob and temporal synchronization of repetitive finger movements. Negative linear relationship between cortical myelination (as indexed by the mean R1 signal) in the precentral hand knob and the coefficient of variation of the intermovement interval during the visuomotor abduction task. ***A***, ***C***, The same negative relationship became evident when the task was performed with the left index finger (***A***) or little finger (***C***). Open and closed black circles indicate subjects with hotspots located in the posterior lip of precentral crown and the top of the precentral crown, respectively. ***B***, ***D***, Surface-rendered voxel-wise correlation maps indicating a negative relationship between precentral cortical myelination and the coefficient of variation of the intermovement interval during the visuomotor abduction task and for the index finger (***B***) and little finger (***D***). Statistical maps are thresholded at *p*_uncorrected_ < 0.01 for illustrative purposes.

Precentral myelination did not only predict task-related BOLD signal changes in the precentral motor hand knob, but also the temporal reliability of movement repetition in the visuomotor synchronization task (Fig. 6) We found that participants more precisely synchronized their finger movements to the external pace, the higher the precentral myelin content. There was a significant negative linear relation between precentral cortical myelination, as indexed by regional R1 value, and the mean CV of movement repetition rate for both fingers (index finger: *r* = −0.619, *p* = 0.004; little finger: *r* = −0.684, *p* < 0.001; Fig. 6*A*,*C*). This indicates that individuals with a higher degree of cortical myelination of the precentral gyrus showed a more regular timing of repetitive finger movements with less intertrial variation of the between-movement interval. Surface-rendered maps of this relationship at voxel levels yielded several clusters that were mainly located in, but not limited to, the precentral crown (Fig. 6*B*,*D*). Follow-up analyses based on the mean R1 signal from the two Pre-CG ROIs revealed that the myelin content in the rostral part (index finger: *r* = −0.601, *p* = 0.004; little finger: *r* = −0.662, *p* = 0.001) and the caudal part of the motor hand knob (index finger: *r* = −0.590, *p* = 0.006; little finger: *r* = −0.626, *p* = 0.003) scaled negatively with the mean CV of movement repetition rate for both fingers.

We also performed exploratory analyses in which we tested for linear relationships between hotspot rostrality and functional motor readouts (FDI muscle: *r* = 0.450, *p* = 0.031; ADM muscle: *r* = 0.525, *p* = 0.008). As for the mean R1 signal in precentral gyrus, we found that interindividual variations in BOLD signal change and movement repetition rate also scaled linearly with individual hotspot rostrality, showing that this functional TMS readout also reflects interindividual differences in cortical motor function at the level of regional neural activity and timing performance (FDI muscle: *r* = −0.617, *p* = 0.0013; ADM muscle: *r* = −0.619, *p* = 0.0013).

### Hotspot location is related to induced electrical field magnitude in precentral gyrus

**Figure 7.**
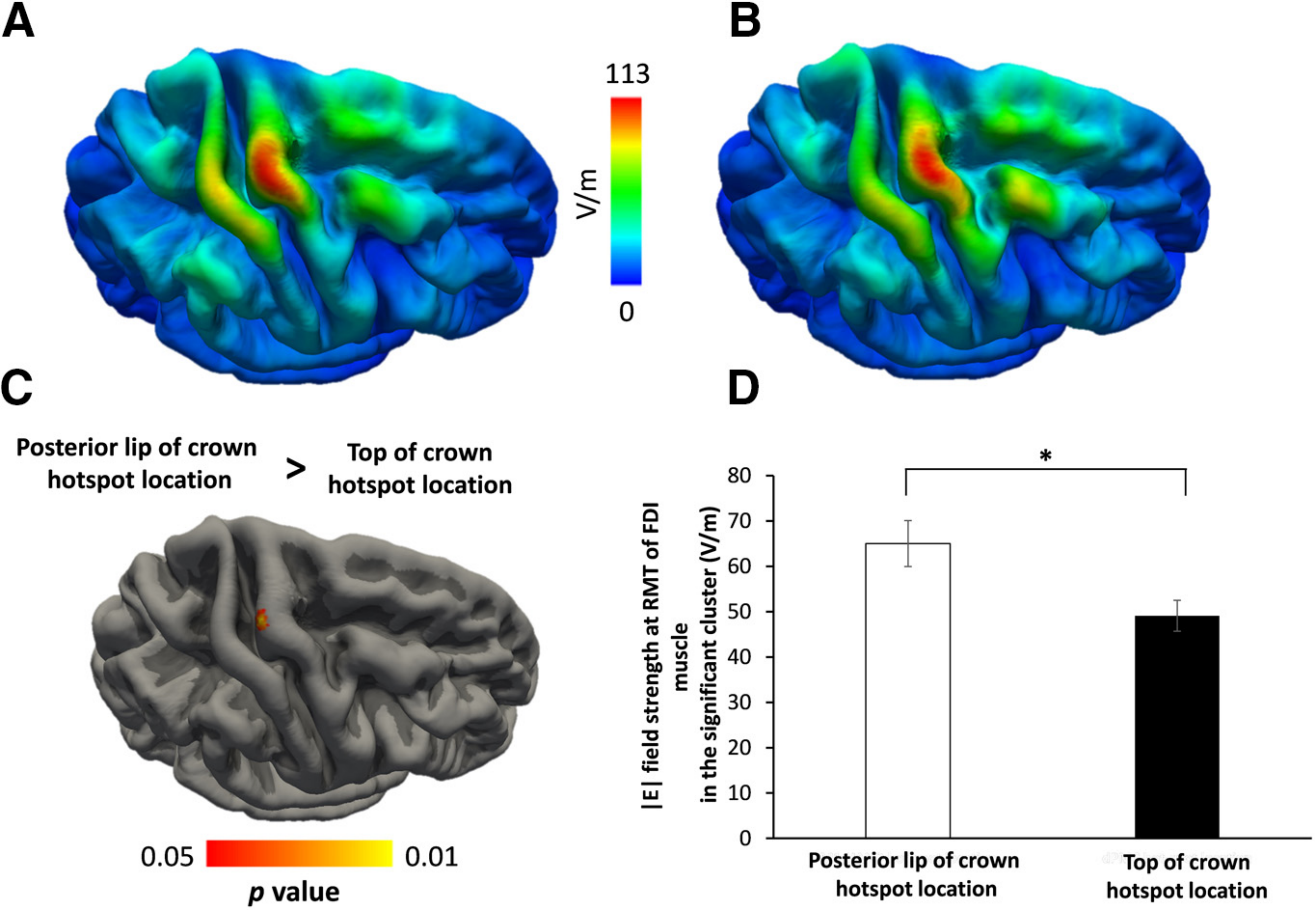
Simulations of the electric field strength induced by TMS at motor hotspot locations. ***A***, ***B***, Color-coded surface-rendered group maps for participants with preferential hotspot locations in the posterior lip of the precentral gyrus (***A***) or at the top of the precentral crown (***B***). ***C***, The surface-based statistical map shows a single cluster in the posterior lip of the precentral gyrus where participants with preferential hotspot location in the posterior lip of the pre-central gyrus crown display a higher electrical field strength than participants with a more rostral location of motor hotspot. ***D***, Between-groups difference of the mean electric field strength extracted from the significant cluster in the posterior lip region. Mean electrical field strength in this cluster is higher in subjects with a more posterior motor hotspot location in the precentral crown (white column) than in subjects with a more rostral motor hotspot location (black bar; *t_(16.7)_ = 2.604, *p* = 0.019; unpaired *t* test).

Since all participants underwent structural T1-weighted and T2-weighted MRI scans, we were able to simulate the electric fields that were generated in the right precentral gyrus by the TMS pulse at the individual precentral hotspot as determined in the preparatory TMS session. We first created a group map of the electrical field distributions for the motor hotspot locations. Electric field strength was high in the precentral crown and weak in the sulcal parts of the precentral gyrus (caudal Pre-CG ROI, 71.14 ± 6.57 V/m; vs rostral Pre-CG ROI, 96.4 ± 6.44 V/m; paired t test: *t* (23) = −8.825, *p* < 0.001; Fig. 2*E*). We statistically compared the TMS-induced electrical field distributions in the right precentral hand knob between the groups with a rostral or a caudal hotspot location in the main experiment (Fig. 7). The color-coded surface-rendered maps of the regionally induced electrical field confirmed that the average of electric field strength was maximal in the precentral crown corresponding to the precentral hand knob with additional local peaks with lower intensity in neighboring gyral crowns (Fig. 7*A*,*B*). Although the spatial distribution of the TMS-induced electrical fields was similar in the right precentral hand knob, a between-group comparison revealed higher electrical field magnitudes in the posterior lip region in the group having a more caudal hotspot location in the shape-based TMS mapping experiment (Fig. 7). The between-group difference in electrical field magnitude peaked at the *x*-, *y*-, and *z*-coordinates (34, −20, and 65, respectively) corresponding to the posterior lip region of the precentral gyrus. The group with a more rostral motor hotspot did not display any clusters in the precentral gyrus where the induced electrical field was higher than in subjects with a more caudal hotspot location. Hence, electrical field strength did not differ between groups in the anterior lip region of the precentral gyrus. In a follow-up analysis, we tested whether the individual E-field strength in precentral gyrus scales would scale with precentral cortical myelin content. We found no cluster in the precentral gyrus showing a significant linear relationship between individual E-field strength and cortical myelin content.

## Discussion

In healthy human volunteers, we probed the regional structure and function of the right precentral motor hand knob with TMS, structural MRI, and functional MRI. Our multimodal brain-mapping approach revealed a link among cortical myelin content, functional corticomotor representations, and dexterous motor control. A higher myelin content of the precentral motor hand knob was associated with more rostral corticomotor presentations, as revealed by shape-informed TMS mapping, with stronger task-related activation during task-based fMRI, and a higher precision of movement timing during a visuomotor synchronization task.

### Relationship between precentral myelin content and rostrality of corticomotor muscle presentations

The spatial location of the motor hotspot, defined as the optimal scalp position where TMS evokes a contralateral motor response, varied across individuals along an anterior–posterior axis. In contrast to standard grid-based mapping, our sulcus-aligned mapping procedure secured the idea that TMS at each stimulation site produced a consistent current direction in the most strongly stimulated part of the precentral hand knob regardless of the individual folding pattern. Extending previous work, we found that a more rostral hotspot location in the precentral crown was associated with a longer corticomotor MEP latency. In individuals in whom the motor hot spot was spatially more distant from the central sulcus, stimulation also occurred functionally more upstream from the corticospinal motoneurons, resulting in longer latencies. In other words, “spatial” rostrality of the individual motor hotspot was mirrored by a “temporal” or “functional” rostrality of the motor hotspot. MRI mapping of the R1 signal revealed that the spatiotemporal hotspot rostrality had a structural correlate in the precentral hand knob. The degree of precentral myelination, reflected by the mean regional R1 signal, but not the cortical thickness, scaled with interindividual differences in spatiotemporal hotspot rostrality.

### TMS of the motor hand knob is spatially biased toward the superficial crown-lip region

The human M1_HAND_ consists of a posterior (or caudal) region that is located in the depth of the anterior sulcal wall (referred to as BA4p) and an anterior (or rostral) region (referred to as BA4a) that is located more superficially in the anterior wall and may expand into the superficial crown-lip region (Geyer et al., 1996, 2000). Retrograde tracing studies in the macaque monkey identified the caudal portion of M1 in the anterior bank of the central sulcus as the main precentral source of cortico-motoneuronal pyramidal output neurons (Rathelot and Strick, 2006, 2009). The number of fast-conducting corticospinal pyramidal output neurons with direct synaptic connections to the cervical motoneurons are mainly found in the caudal “new” portion of the M1_HAND_ corresponding to area BA4p in humans (Rathelot and Strick, 2006, 2009). Since the vast majority of fast-conducting corticospinal pyramidal output neurons originate from the caudal M1_HAND_ in the depth of the central sulcus, these neurons, but also the axons of other interneurons and pyramidal cells in caudal M1_HAND_ (BA4p), are “out of reach” for the TMS-induced electrical field, which primarily targets the superficial crown-lip region in the precentral gyrus. We therefore argue that cortical regions that are located more superficially and are functionally more upstream, such as the rostral M1_HAND_ (BA4a) and the caudal part of PMd, are the primary excited cortical areas when TMS is applied to the motor hot spot, at least at stimulation intensities just above corticomotor resting threshold. The primary stimulation of superficial precentral areas then causes a transsynaptic (indirect) excitation of fast-conducting corticospinal output neurons via corticocortical axonal projections from anterior M1_HAND_ and the caudal part of PMd to the posterior M1_HAND_ (Siebner, 2020).

A recent biophysical modeling study identified axonal terminations in the crown-lip region of the precentral gyrus, which are aligned to the locally induced E-field direction, as primary target structures for TMS-induced neuronal excitation (Aberra et al., 2020). Efficient excitation of axonal terminations within the precentral crown-lip region will lead to transsynaptic excitation of other cortical neurons in this area (i.e., axonal-termination hypothesis). The highly synchronized neuronal activity in the stimulated crown-lip region may then spread to deeper parts of the precentral cortex (especially the caudal M1_HAND_) in the sulcal wall via corticocortical axonal fibers. Alternatively, it is possible that the induced electrical field primarily stimulates the bends of juxtacortical corticocortical axons that originate in the precentral crown and project into the depth of the sulcus where the bulk of the fast-conducting corticospinal motoneurons are located (i.e., axonal-bend hypothesis). Electric field modeling suggests that the field strength in juxtacortical white matter of the precentral crown is indeed higher than the field strength in the overlying gray matter (Thielscher et al., 2011), and axonal bends have been identified as putative stimulation sites based on morphologically simplified neural models (Salvador et al., 2011).

### Direction-specific target engagement of axonal structures in the precentral crown

We used biphasic TMS pulses for shaped-informed TMS mapping as in our previous studies (Raffin et al., 2015; Dubbioso et al., 2017; Raffin and Siebner, 2019). The biphasic pulse produced two neurobiologically relevant currents in the precentral gyrus: the first phase of the biphasic pulse produced an A–P directed current, while the second phase caused a current with a P–A direction. The second P–A component is neurobiologically most effective given the longer duration and larger area under the curve, but the first A–P component may also induce action potentials and contribute to the MEP. The direction-dependent effects of the A–P and P–A components of the biphasic pulse may determine the anterior–posterior hot spot location. In their modeling study, Aberra et al. (2020) showed that a monophasic TMS pulse producing an A–P directed current in the precentral gyrus resulted in an anterior shift of activation of axon terminals of pyramidal neurons in layer 2 or 5 compared with a monophasic TMS pulse causing a current in the opposite (P–A) direction. Interestingly, this pattern also emerged albeit to a lesser extent, when simulating the precentral crown with a biphasic pulse configuration (Aberra et al., 2020). This implies that a P–A directed current will result in lower thresholds for efficient axonal depolarization in the posterior lip region, while an A–P directed current will result in lower excitation thresholds in the anterior lip region of the crown. The biophysical properties of biphasic TMS may account for the present results. The A–P component of the TMS pulse may have been more relevant for overall stimulation of axonal structures in the precentral crown in individuals with a more anterior precentral motor hotspot location: in these individuals, the A–P current may have been more effective to induce suprathreshold depolarization of axonal terminations in the anterior lip region. Conversely, the depolarizing effects of the P–A current may be more prevalent in individuals with a more posterior motor hotspot location. In these individuals, a preponderant excitation of axonal terminations in the posterior lip region may have shifted the precentral motor hotspot posteriorly. This explanation is corroborated by the simulations of the TMS-induced electrical field at the hot spot location. Here, TMS induced a stronger electrical field in the posterior lip of the precentral crown only in individuals in whom sulcus-shaped TMS mapping revealed a caudal posterior precentral motor hotspot.

In addition to intracortical excitation of axon terminals, a biphasic TMS pulse may effectively depolarize longer-range juxtacortical axons at their bends in the subcortical white matter within the precentral crown-lip region. Since the depolarizing effect of the electrical field on the axonal bend depends on its orientation relative to the E-field, the A–P and P–A directed currents may result in spatially distinct hotspots. The A–P directed current preferentially stimulates axonal bends in more anterior (rostral) locations within the precentral crown, whereas the P–A directed current may preferentially stimulate axonal bends in more posterior (caudal) locations in the precentral crown. The capability to excite these axon bends may differ across individuals and, thus, contribute to interindividual variations in motor hotspot location.

### Positive linear relationship between spatial and temporal hot spot rostrality

In our study, participants with a more rostral hotspot had longer corticomotor latencies than those with a more caudal hot spot location. The interindividual variation in corticomotor latencies may be caused by the interindividual variation in preferential A–P stimulation in the precentral crown. According to the axonal-termination hypothesis, a preferential stimulation of axonal terminations in the anterior lip region (A–P current) or posterior lip region (P–A current) of the precentral crown will alter the corticocortical conduction time from the precentral crown to the caudal M1_HAND_ (BA4p area) in the depth of the central sulcus. This explanation is in good agreement with previous TMS studies on the impact of a current reversal for monophasic pulse configurations: reversing the current flow from P–A to A–P in the precentral crown also results in longer corticomotor latencies and higher corticomotor thresholds (Hamada et al., 2013), and stronger susceptibility to variations in pulse length (D'Ostilio et al., 2016). Furthermore, MEP latency after A–P TMS was found to correlate with functional connectivity between M1 and a network involving cortical premotor areas (Volz et al., 2015).

The axonal-bend hypothesis may also explain the observed variation in corticomotor latency. The folding of the cortex at the gyral crown alters the curvature of axonal bends in the juxtacortical part of the anterior and posterior lip region, which renders these spatially segregated segments of the same axons more or less susceptible to the anterior–posterior phase of the biphasic TMS pulse. Accordingly, the A–P and P–A current components of the biphasic pulse may stimulate the same juxtacortical corticocortical axons at more anterior or posterior positions in the precentral crown. In humans, TMS activates neural elements having time constants determined from strength–duration curves of ∼200 µs (Barker et al., 1991; Peterchev et al., 2013; D'Ostilio et al., 2016) with conduction times comparable to peripheral motor axons. A very conservative estimate would be that these axons have conduction speeds of ∼10 m/s (West and Wolstencroft, 1983; Firmin et al., 2014). In this case, the observed MEP latency differences would correspond to a traveled distance of >12 mm, which is in the range of the observed variations of the hotspot in caudal–rostral direction. For a slightly less conservative estimate of conduction speeds of ≥20 m/s, the observed MEP latency differences are too long to be explained merely by the stimulation of two positions of the same axons anymore, but rather favors the targeting of different neural populations. Finally, the axon diameter distribution of corticocortical projection fibers within the precentral gyrus may have contributed to the between-subject variations in corticomotor latency (Liewald et al., 2014). The larger the corticocortical axons and the thicker their myelin sheets, the faster the signal transmission from the primary site of stimulation in the precentral crown to the fast-conducting corticospinal output neurons originating from the caudal M1_HAND_ (BA4p area) in the depth of the central sulcus.

### Positive linear relationship between hotspot rostrality and precentral myelination

Our results establish a link between the myelination of the precentral hand knob and motor hot spot location, showing that the cortical myelin content scaled with individual motor hotspot rostrality. The axonal-termination hypothesis of TMS-induced cortex stimulation can account for this positive relationship. Stronger myelination of axons may lower the excitation threshold of axonal terminals in the anterior and posterior crown-lip region of the precentral gyrus to fire action potentials in response to TMS. This may be particularly relevant for the efficacy of the A–P component of the biphasic pulse, which is less efficient than the P–A component. If axon terminations are more myelinated in the anterior lip region of the central gyrus, they will become more susceptible to the depolarizing effect of the A–P directed current. The A–P directed current will contribute more to the overall stimulation of axonal terminations in the precentral crown and shift the motor hotspot location to a more anterior location. Conversely, the motor hotspot will be located more caudally if the P–A component makes the strongest contribution to overall axonal depolarization in the precentral crown.

The axonal-bend hypothesis of TMS-induced cortex stimulation can also account for the positive relationship between hotspot rostrality and the degree of precentral myelination. If juxtacortical axonal bends in the anterior lip region are more strongly myelinated, they may be more easily depolarized by the A–P directed current. A relatively stronger contribution of the A–P directed current to the overall excitation of axonal bends in the precentral crown will result in an anterior shift of the motor hotspot.

### A macroanatomic perspective on spatiotemporal hotspot variability

Do the interindividual differences in spatiotemporal hotspot rostrality indicate that TMS preferentially excites cytoarchitectonically different cortex regions? The rostral M1_HAND_ (BA4a) and caudal PMd in the precentral crown-lip regions are the primary target regions, when TMS is applied at the precentral motor hot spot (Aberra et al., 2020). The border between rostral M1_HAND_ and caudal PMd is not sharply demarcated, but is smooth, and the transition may vary considerably along the anterior–posterior axis in the precentral gyrus (Geyer et al., 1996, 2000). In some individuals, the transition zone between M1_HAND_ and PMd extends to the precentral crown-lip region, making the superficial part of the rostral M1_HAND_ a primary target for TMS in individuals with a caudal motor hot spot location. Therefore, one should be cautious to conclude that in individuals with an anterior or posterior precentral hot spot, TMS preferentially stimulates more rostral or caudal parts of PMd, respectively. It is also possible that TMS preferentially stimulates the most anterior part of the rostral M1_HAND_ in individuals with a posterior precentral motor hotspot.

In macaque monkeys, intracortical electrical stimulation revealed that both the rostral (old) and caudal (new) part of M1 send slowly conducting monosynaptic corticospinal projections to the cervical motoneurons, while only the caudal (new) M1 hosted pyramidal cells with fast monosynaptic corticospinal projections to the cervical spinal motoneurons (Witham et al., 2016). In persons with a posterior precentral hotspot, the slowly conducting monosynaptic corticospinal projections to the cervical motoneurons may be readily stimulated by TMS via excitation of local axonal terminals, if the rostral M1_HAND_ extends rostrally into the posterior crown-lip region.

### Linking precentral myelination and motor function

Regional myelination showed a positive linear relationship with motor activation in the precentral hand knob during task-based MRI in the anterior lip region of the precentral crown. The stronger task-related engagement of the right caudal PMd in individuals with a higher precentral myelin content is in good agreement with previous work showing that the PMd plays a prominent role in visuomotor integration (Jäncke et al., 2000; Sugiura et al., 2001; Cerasa et al., 2005; Chouinard and Paus, 2006; Witt et al., 2008; Hardwick et al., 2015). We also identified several clusters in the rostral and caudal part of the precentral motor hand knob where a higher cortical myelin content was associated with a higher degree of temporal regularity during the finger-tapping task. Our motor task probed temporal aspects of dexterous motor control, because participants had to match the timing of tapping to the regular 1 Hz pace given by an external cue. Individuals with a higher precentral myelin signal were better at minimizing intertrial variations between consecutive movements. We therefore argue that a higher degree of myelination of the precentral gyrus enabled a more precise synchronization of finger tapping with the regular pace provided by the visual cue.

How does cortical myelination support the integration of neuronal activity within functional brain networks? Structural MRI studies have linked cortical myelination to intrinsic functional connectivity (Huntenburg et al., 2017) and task-related functional activity in unimodal cortical areas, including the visual cortex (Sánchez-Panchuelo et al., 2012; Sereno et al., 2013), auditory cortex (Dick et al., 2012; Kim and Knösche, 2016), and sensorimotor cortex (Glasser et al., 2016; Kuehn et al., 2017). Axonal myelination enables fast signal propagation, synchronizes neural activity, and determines the properties of neuronal activity subserving temporal coding, such as spike latency and interspike interval (Seidl et al., 2010; Pajevic et al., 2014; Ford et al., 2015; Timmler and Simons, 2019). Our results lend further support to the notion that a high degree of myelination in adult neocortex is critical to fast and temporally precise regional neuronal processing, suggesting that a high degree of precentral myelin content enables higher temporal precision during dexterous hand use.

### Conclusion

We provide first-time evidence for behaviorally relevant structural and functional phenotypic variation in the crown of the human precentral motor hand knob. Linking variations in regional brain structure and function, and regional excitability and dexterity, our results corroborate the functional relevance of cortical myelin for cortical function and related behavior.

Our results are also of relevance to the research community that uses TMS of the human M1_HAND_ to study motor cortex plasticity. Defining the individual precentral motor hotspot location is the standard method for spatial targeting of human M1_HAND_ (Groppa et al., 2012; Rossini et al., 2015). Our work questions the assumption that hotspot-based targeting can secure a comparable stimulation of the precentral motor hand knob across subjects. In recent years, it has been emphasized that the plasticity-induced effects of repetitive TMS targeting M1_HAND_ suffers from substantial interindividual variability (López-Alonso et al., 2014; Ziemann and Siebner, 2015). Interindividual differences in motor hotspot rostrality may constitute a major contributing factor to interindividual differences in the aftereffects on corticomotor excitability. Since interindividual differences in hotspot rostrality are associated with different microstructural properties in terms of cortical myelination, it is possible that individuals with a more rostral or caudal motor hotspot may express different forms of precentral motor plasticity.
